# An Address

**Published:** 1864-02

**Authors:** Henry S. Chaw


### AN ADDRESS.

Written for the Iowa Dental Society, Jan. 15th, 1864.—By Henry S. Chaw, M.D.

*Gentlemen of the Dental Society:*—It is with profound regret, that I announce my inability to be present on so interesting an occasion. And I should consider it a neglect of duty, did I not let you know that I am with you, heart and soul, in the great objects of this Association.

Our calling is a noble one, and will compare favorably in dignity and usefulness with all others. Even the physician, heaven-born does not approach the Dentist in the amount of suffering yearly prevented and relieved.

Few persons, in these days, arrive at the age of twenty without experiencing the horrors of "tooth ache." Look where you will, in city or country, in the New or Old world, *everywhere*, one sees the evidences of decay in those beautiful organs, which, from time immemorial, have given to the Poet inspiration, and to female beauty one of its greatest charms.

How great, then, the responsibility of the dentist! How important that he should be master of his profession! and especially of that part called Surgical! The preservation of the natural teeth should be his highest ambition.

On the successful accomplishment of this, he shows himself *superior*, both in *mind* and *manipulation*, to the mere maker of artificial substitutes. Notwithstanding these are furnished rivalling Nature in beauty and usefulness, it is lowering the profession to a mere *trade*, to encourage their adoption instead of insisting on the paramount importance of the preservation of the natural organs.

And let me assure you, Gentlemen, that when it is thus degraded, its honor and profit are forever lost.

The dentists of Iowa have an important work to perform, on the faithful execution of which depends the standing of the profession, not only for the present, but for the *future*. And *what* a future! Look abroad, over the beautiful prairies of our State, with a wealth of soil unsurpassed in the world, and capable of supporting a population of twenty millions! see them thickly dotted over with neat farm-houses, thriving villages and great towns, occupied by a happy and virtuous people!—and can you fail to realize the powerful influence, for weal or woe, *you* have over the comfort and happiness of that people. Let us educate ourselves up to the full standard of honorable, conscientious and capable practitioners in our profession, and it will be well for us, our successors and future generations.

There is a work which is much neglected among us. I mean the education of the public, in regard to our profession. They should be taught how best to preserve their natural teeth, as far as their own daily care is concerned, and also the best modes of dental practice. This should be done, not only as occasions offer in our offices, but through our local newspapers and by public lectures. The more people know of the subject, the better will they appreciate the value of their teeth, and the more willingly they will pay for first-class operations.

I believe the majority of our members are conscientious in giving professional advice. This is a trait which the public will soon discern in the Dentist, and it will redound to his own interest, as well as honor the whole fraternity. And let us not think that we are under no obligations to the profession at large. By becoming its members, we virtually bind ourselves to give to our patients the best skill which we can by constant education acquire, and to add as much to the sum-total of dental knowledge as we can possibly contribute.

Therefore we should not only assemble in conventions and freely contribute our "mites," but we should, through the dental journals, do the same. And what a noble work those dental journals are doing! Well do I remember how narrow-minded and illiberal many practitioners were twenty years ago, when (I think) there was but one publication of the sort in the United States. Now we meet as brothers and *have no scouts.* Thank God for *Progress.*

Finally, Brethren, let us all follow the "Golden Rule," "to do to others as we should wish them to do to us." Fraternal courtesy should be practised by us all, and held in high estimation. The churlishness of one toward us should not prevent us from treating *him* with *kindness.* How often kindness wins over the most intractable natures.

Farewell, my Brothers; God bless you all; and may your meetings be pleasant and useful.

